# Syntheses and crystal structure of 4-[(pyridin-3-yl)diazen­yl]morpholine and 1-[(pyridin-3-yl)diazen­yl]-1,2,3,4-tetra­hydro­quinoline

**DOI:** 10.1107/S2056989023000129

**Published:** 2023-01-10

**Authors:** Seynabou Sokhna, Insa Seck, Ibrahima El Hadj Thiam, Marc Presset, Samba Fama Ndoye, Lalla Aïcha Ba, Issa Samb, Simon Coles, James Orton, Matar Seck, Erwan Le Gall, Mohamed Gaye

**Affiliations:** a Laboratoire de Chimie Organique et Thérapeutique, Faculté de Médecine, de Pharmacie et Odontologie, Université Cheikh Anta, Diop de Dakar, BP 5005, Dakar-Fann, Senegal; b Laboratoire de Chimie de Coordination Organique, Département de Chimie, Faculté des Sciences et Techniques, Université Cheikh Anta Diop, Dakar, Senegal; c UMR 7182 - ICMPE - Institut de Chimie et des Matériaux Paris Est, Thiais, France; d Université Amadou Mahtar MBOW, BP 45927, Dakar Nafa VDN, Dakar-Fann, Senegal; e Equipe de Recherche Chimie Organique et Thérapeutique (ECOT), Université Alioune, Diop de Bambey, Senegal; f UK National Crystallography Service, School of Chemistry, Faculty of Engineering and Physical Sciences, University of Southampton, Southampton SO17 1BJ, United Kingdom; Universidade de Sâo Paulo, Brazil

**Keywords:** 1,2,3-triazenes, morpholine, 1,2,3,4-tetra­hydro­quinolein, pyridine, crystal structure

## Abstract

Two new heterocyclic 1,2,3-triazenes were synthesized by diazo­tation of 3-amino­pyridine following respectively by coupling with morpholine or 1,2,3,4-tetra­hydro­quinoline. 4-[(Pyridin-3-yl)diazen­yl]morpholine (**I**), C_9_H_12_N_4_O, has monoclinic *P*2_1_/*c* symmetry at 100 K, while 1-[(pyridin-3-yl)diazen­yl]-1,2,3,4-tetra­hydro­quinoline (**II**), C_14_H_14_N_4_, has monoclinic *P*2_1_/*n* symmetry at 100 K. These 1,2,3-triazene derivatives were synthesized by the organic medium method by coupling reactions of 3-amino­pyridine with morpholine and 1,2,3,4-tetra­hydro­quinoline, respectively, and characterized by ^1^H NMR, ^13^C NMR, IR, mass spectrometry, and single-crystal X-ray diffraction. The mol­ecule of compound **I** consists of pyridine and morpholine rings connected by an azo moiety (–N=N–). In the mol­ecule of **II**, the pyridine ring and the 1,2,3,4-tetra­hydro­quinoline unit are also connected by an azo moiety. The double- and single-bond distances in the triazene chain are comparable for the two compounds. In both crystal structures, the mol­ecules are connected by C—H⋯N inter­actions, forming infinite chains for **I** and layers parallel to the *bc* plane for **II**.

## Abstract 2

The title triazene derivatives were synthesized using a diazo­nium inter­mediate that was obtained from 3-amino­pyridine and isoamyl nitrite.

## 1. Chemical context

1,2,3-Triazenes are versatile compounds in preparative chemistry because of their stable and highly modular nature (Patil & Bugarin, 2016 ▸). 1,2,3-Triazene derivatives have been studied for their potential anti­cancer properties (Rouzer *et al.*, 1996 ▸; Connors *et al.*, 1976 ▸), used as a protecting group in natural product synthesis (Nicolaou *et al.*, 1999 ▸) and combinatorial chemistry (Bräse *et al.*, 2000 ▸), incorporated into polymers (Jones *et al.*, 1997 ▸) and oligomer synthesis (Moore, 1997 ▸), and used to prepare heterocycles (Wirschun *et al.*, 1998 ▸). 1,2,3-Triazenes are some of the most important compounds proposed for electrochromic materials that change color in the presence of the missing light in response to electrochemical switching (Monk *et al.*, 2007 ▸). This phenomenon has potential utility in protective eyewear and data storage devices applications (Mortimer, 1997 ▸, 1999 ▸; Argun *et al.* 2004 ▸; Lampert, 1984 ▸). These mol­ecules constitute a unique class of compounds containing three adjacent nitro­gen atoms in an acyclic arrangement (Kimball & Haley, 2002 ▸; Nwajiobi *et al.*, 2022 ▸; Bormann *et al.*, 2022 ▸). 1,2,3-Triazenes can be prepared by diazo coupling between a diazo­nium salt and primary, or secondary amines (Sadtchikova & Mokrushin, 2002 ▸) or Grignard reagents coupled with azides (Kirk, 1978 ▸). The synthesis of this type of compound in water as solvent is one of the most important challenges in green chemistry as the reaction conditions minimize environmental haza­rds and chemical waste (Zhang *et al.*, 2018 ▸). 1,2,3-Triazenes can exist as a mixture of tautomers. The nature of the mixture and equilibrium position can be defined by crystallographic studies. It is in this context that we synthesized two triazene derivatives and determined their structures by XRD. 
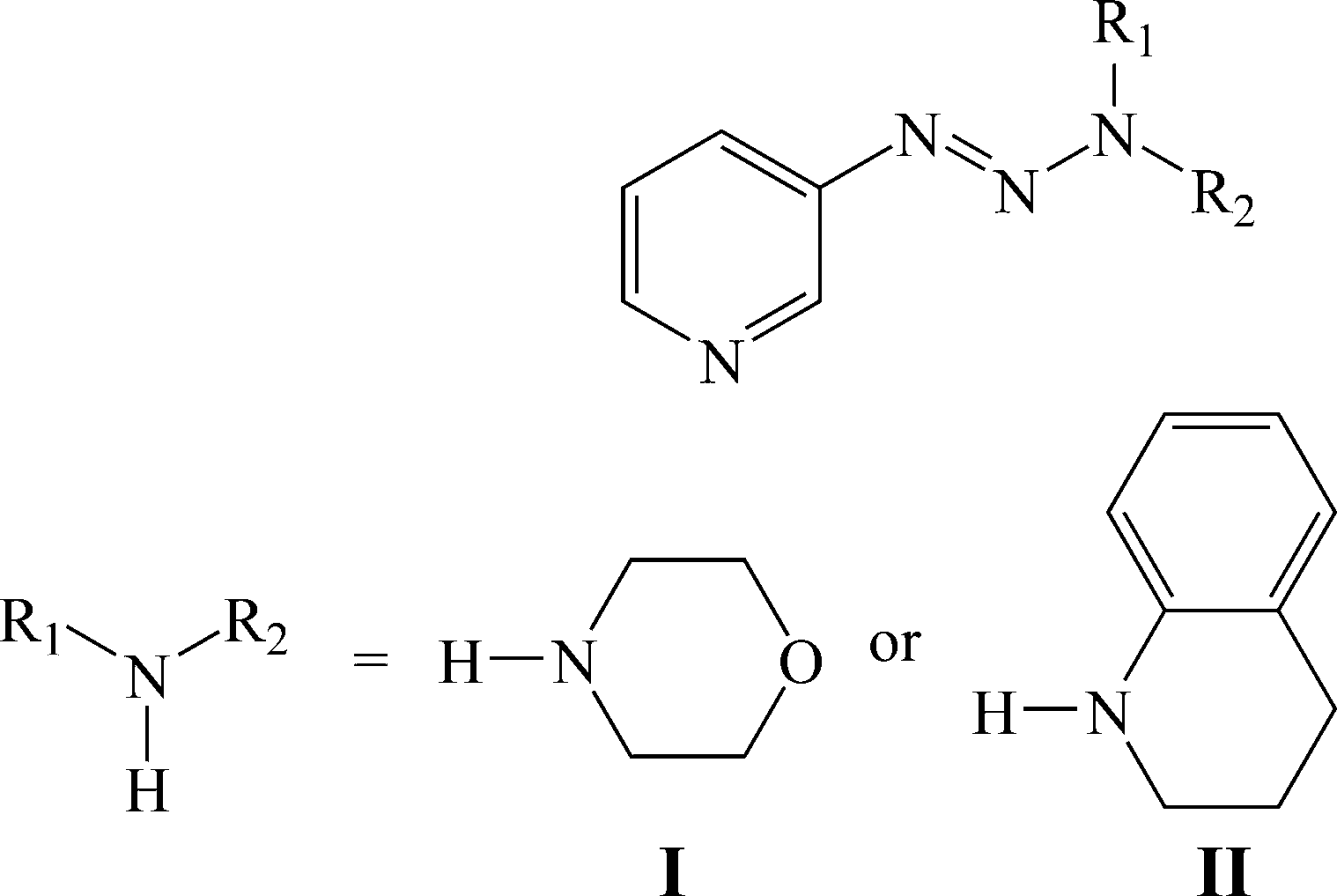


## 2. Structural commentary

**Figure 1:**
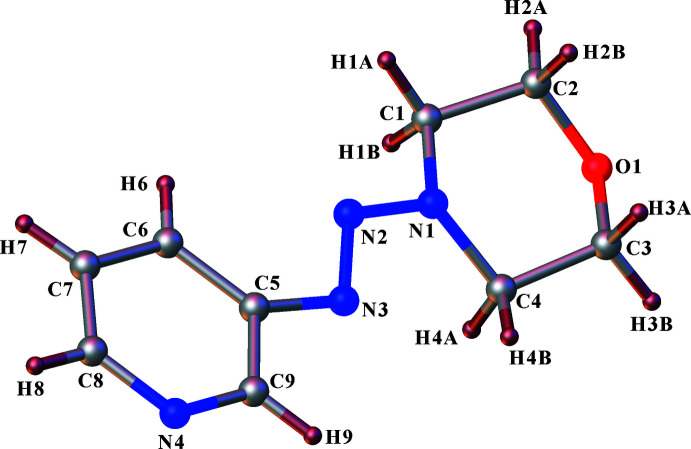
A view of the title compound **I**, showing the atom-numbering scheme. Displacement ellipsoids are plotted at the 30% probability level.

**Figure 2:**
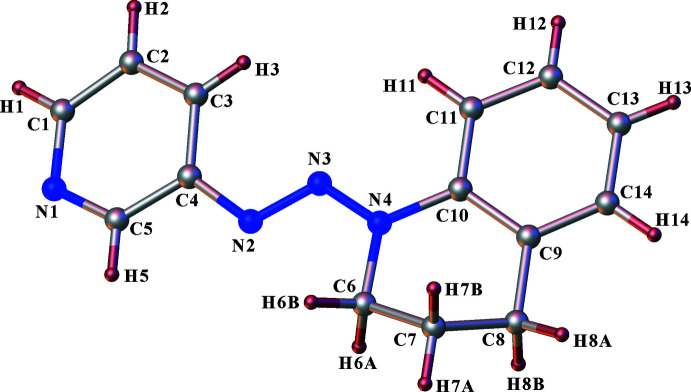
A view of the title compound **II**, showing the atom-numbering scheme. Displacement ellipsoids are plotted at the 30% probability level.

Compound **I** was synthesized *via* reaction of the diazo­nium salt of 3-amino­pyridine and morpholine. The resulting compound was recrystallized from ethanol to yield orange single crystals. Compound **I** crystallizes in the centrosymmetric monoclinic space group *P*2_1_/*c*, with the asymmetric unit consisting of one 1-morpholino-2-(pyridin-3-yl)diazene mol­ecule (Fig. 1 ▸). The mol­ecule consists of six-membered pyridine and morpholine rings connected by an –N=N– moiety through the nitro­gen atom of the morpholine ring and a carbon atom of the pyridine ring. Thus a 1,2,3-triazene moiety (–N=N—N–) is formed in which the double-bond character of the azo moiety is indicated by the bond distance of 1.2640 (12) Å for N2—N3. The bond distance of 1.3350 (11) Å is indicative of single-bond character for N1—N2 moiety. The N2—N3 bond adopts an (*E*)-configuration. The pyridyl group is *trans* with respect to the morpholino group across the N2—N3 bond. The morpholine ring has a chair conformation with N1 and O1 situated, respectively, 0.192 (1) Å to one side of the mean plane through all ring atoms and 0.273 (1) Å to the other. Thus, O1 and N1 atoms are in a *syn* conformation with respect to the C1—C2 link [N1—C1—C2—O1 = 55.81 (11)°] and C3—C4 link [N1—C4—C3—O1 = −54.11 (11)°]. The pyridine ring forms dihedral angles of 8.80 (10) and 12.46 (5)° with the triazene moiety and the mean plane of the morpholine ring, respectively. The C—C bond lengths in the pyridine ring are in the normal range [1.33–1.39 Å]. In fact, the C5—C6 and C8—N4 bond lengths [1.3928 (14) and 1.3351 (15) Å, respectively] are characteristic of a delocalized pyridine ring (Wahedy *et al.*, 2017 ▸). The C—C—C bond angles in the ring measure almost 120°, with a maximum deviation of less than 2°, indicating that the atoms involved are *sp* ^2^-hybridized. All the bond angles involving the morpholine heterocyclic ring atoms, which fall in the range 108.17 (8)–116.08 (8)°, are close of the ideal value of 109° for a perfect tetra­hedral carbon atom, and are indicative of *sp* ^3^-hybridized carbon atoms in the heterocyclic ring. The values of the bond distances in the chain, N3— N2 = 1.2640 (12) Å and N2— N1 = 1.3350 (11) Å, indicate their respective double- and single-bond characters. The N3—N2—N1 angle of 114.09 (8)° confirms the formation of the triazene compound (Fig. 2 ▸).

Compound **II** crystallizes in the centrosymmetric monoclinic space group *P*2_1_/*n*, with the asymmetric unit consisting of one 1-[3,4-di­hydro­quinolin-1(2*H*)-yl]-2-(pyridin-3-yl)diazene mol­ecule. The mol­ecule consists of a pyridine ring and a tetra­hydro­quinoline moiety connected by an azo unit (–N=N–) through the nitro­gen atom of the 1,2,3,4-tetra­hydro­quinoline ring and a carbon atom of the pyridine ring. Thus a 1,2,3-triazene moiety (–N=N—N–) is formed in which the double-bond character of the azo moiety is indicated by the bond distance of 1.2737 (13) Å for N2—N3 while the bond distance of 1.3341 (12) Å shows the single-bond character of N3—N4. The N2—N3 bond adopts an (*E*)-configuration. The pyridyl group is *trans* with respect to the tetra­quinolyl group across the N2—N3 bond. The mean planes of the fused benzene and piperidine rings are not coplanar and form a dihedral angle of 10.79 (5)°. The pyridine ring forms dihedral angles of 12.12 (10), 22.07 (5) and 25.72 (5)° with the triazene moiety, the benzene ring and the piperidine ring, respectively. In the fused piperidine ring, two types of hybridized atoms exist as shown by the different angle values. The angles whose vertices are C9, C10 and N4 are in the range 118.39 (10)–120.41 (10)°, close to the ideal angle of 120° for *sp* ^2^-hybridized atoms. The angles whose vertices are C6, C7 and C8 are in the range 109.95 (9)–110.68 (13)°, close to the ideal angle of 109° for *sp* ^3^-hybridized atoms.

## 3. Supra­molecular features

**Figure 3:**
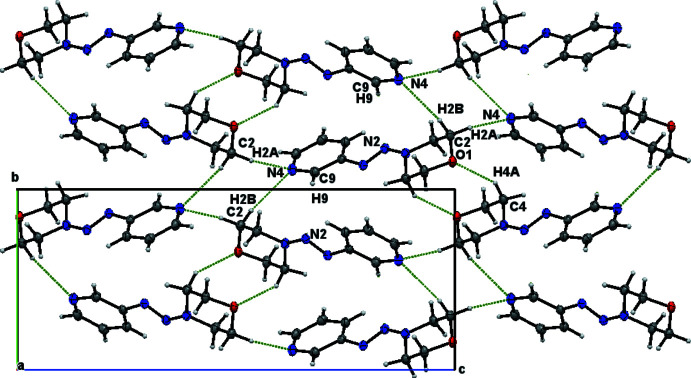
Infinite chains of compound **I** parallel to the *a* axis.

**Figure 4:**
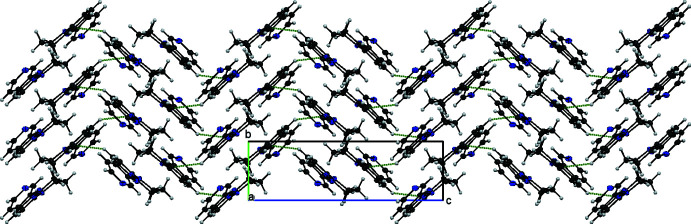
Layers of compound **II** parallel to the *bc* plane.

The the crystal of **I**, non-classical C—H⋯N interactions link the molecules into chains: C3—H3*A*⋯N2^iii^ bonds form chains parallel to the *a* axis, C2—H2*B*⋯N4^ii^ and C7—H7⋯N3^iv^ bonds form chains parallel to the *b* axis and C2—H2*A*⋯N4^i^ bonds form chains parallel to the *c* axis (Table 1 ▸, Fig. 3 ▸). In the crystal of **II**, C12—H12⋯N3^i^ inter­actions link the mol­ecules, forming layers in the *bc* plane (Table 2 ▸, Fig. 4 ▸).

## 4. Database survey

A search of the CSD database (Version 5.43, November 2021; Groom *et al.*, 2016 ▸) gave 48 hits for compounds including morpholino 1,2,3-triazene derivatives similar to compound **I**. Three hits of compounds including the tetra­hydro­quinoline triazene moiety as in compound **II** were found: TADLOB (Huang *et al.* 2010 ▸), VAQMAC and VAQMEG (Katritzky *et al.*, 2003 ▸). Aryl­morpholino 1,2,3-triazenes have structural characteristics like those of compound **I** and contain a 1,2,3-triazine unit consisting of three consecutive conjugated nitro­gen atoms, as seen in **I**. Examination of the structure of EMUDEX (Lee *et al.*, 2016 ▸) suggests that a degree of π-delocalization across the linear triazene moiety of **I** was observed. The N2—N3 double-bond distance of 1.2679 (13) Å and the N1—N2 single-bond distance of 1.3501 (12) Å in EMUDEX are in accordance with those reported for OFUBUO (Mukai *et al.*, 2013 ▸), EZEXEN (Gholivand *et al.*, 2010 ▸), FUZLUI (Pye *et al.*, 2010 ▸). The structures of HAHQOZ (Johnson *et al.* 2016 ▸), HUHGEZ (Isovitsch & Fronczek, 2020 ▸), IJEVUR (Gangwar *et al.*, 2021 ▸), OPAVUX (Chin *et al.*, 2011 ▸) and RUJQIX (Chin *et al.*, 2009 ▸) feature similar inter­molecular hydrogen-bonding inter­actions to those in **I**, resulting in supra­molecular networks.

## 5. Synthesis and crystallization

**Figure 5:**
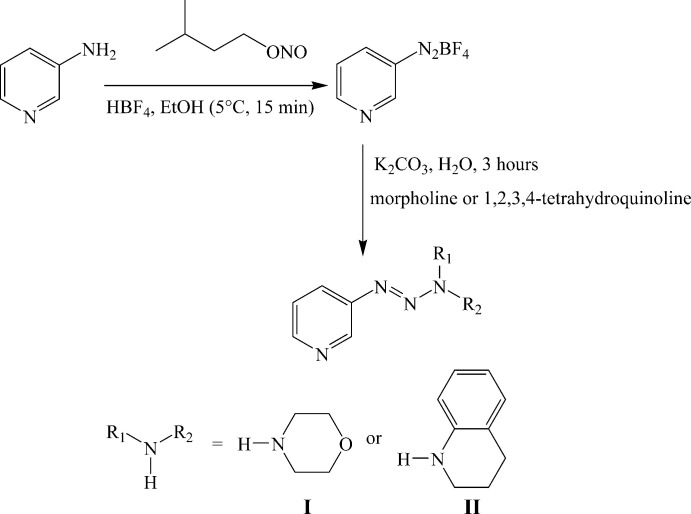
Reaction scheme.

Several methods are known for the synthesis of 1,2,3-triazenes, but the most known is the diazo-coupling method where the diazo­nium salt is formed by the action of NaNO_2_ in an acid medium on a primary amine and coupling of this salt with a primary or secondary amine. In this part of the work, a certain number of difficulties were encountered, in particular concerning the solubility of the synthesized 1,2,3-triazenes in the solvents used for analysis (CDCl_3_ and acetone-*d*_6_). Known by the strong presence of a dipole moment, the analysis of these compounds requires the use of very polar solvents such as DMSO-*d*_6_ or MeOD. The compounds were prepared according to the reaction sequence presented in Fig. 5 ▸. We tried several methods for the synthesis of diazo­nium salts of amino­pyridine derivatives. Finally, we succeeded in obtaining the diazo­nium salt of 3-amino­pyridine using isoamyl nitrite instead of sodium nitrite and ethanol as solvent with good yield. We witnessed an explosion of this salt because of its instability. In a 100 mL flask, 3-amino­pyridine (5 mmol), ethanol (3 mL), HBF_4_ acid (50%, 1.5 mL) and isoamyl nitrite (5 mmol) were added. The mixture was kept under stirring for 15 min at 268 K. To this solution containing the diazo­nium salt, morpholine or 1,2,3,4-tetra­hydro­quinoline (5 mmol) in water (5 mL) was added and the mixture was stirred for 1 h at 273 K. A solution of potassium carbonate in water (5 mL) was added to the flask and the reaction kept under stirring for 3 h at room temperature. The resulting product was extracted with ethyl acetate, dried with Na_2_SO_4_, filtered and evaporated. Compounds **I** and **II** were obtained in a crystalline form with this synthetic method.

Compound **I.** Yield: 72%. Orange crystal, m.p. 356–358 K, HPLC purity: 99.67%. ^1^H MNR (400 MHz, δ (ppm), DMSO-*d*_6_): 8.59 (*d*, *J* = 2.5 Hz, 1 H), 8.40 (*dd*, *J* = 4.7, 1.7 Hz, 1 H), 7.74 (*d*, *J* = 8.3 Hz, 1 H), 7.39 (*dd*, *J* = 8.4, 4.7 Hz, 1 H), 3.78 (*s*, 8 H). ^13^C MNR (100 MHz, δ (ppm), DMSO-*d*_6_): 147.41, 146.07, 143.68, 126.44, 124.3. MS (ESI) (*m*/*z*, %): 194.25 (12), 193.2 ([*M*+1], 100).

Compound **II** Yield: 28%. Orange crystal, m.p. 343–350 K, HPLC purity: 99.82%. ^1^H MNR (400 MHz, δ (ppm), CDCl_3_: 8.86 (*s*, 1 H), 8.45 (*d*, *J* = 4.8 Hz,1 H), 7.90 (*dd*, *J* = 8.4, 2; 7 Hz, 1 H), 7.83 (*d*, *J* = 8.3 Hz, 1 H), 7.34 (*dd*, *J* = 8.2, 4.8 Hz, 1 H), 7.30–7.23 (*m*, 1 H), 7.17 (*d*, *J* = 7.5 Hz, 1 H), 7.05 (*d*, *J* = 8.2, 1 H), 4.13 (*t*, *J* = 5.9 Hz, 2 H), 2.85 (*t*, *J* = 6.1 Hz, 2 H), 2.21–2.10 (*m*, 2H). ^1^C MNR (100 MHz, δ (ppm), CDCl_3_): 147.41, 146.42, 144.76, 139.96, 128.93, 127.52, 126.77, 126.14, 123.75, 123.16, 115.47. MS (ESI) (*m*/*z*, %): 240.29 (21), 477.29 (7), 239.17 ([*M*+1], 100).

## 6. Refinement

Crystal data, data collection and structure refinement details are summarized in Table 3 ▸. All H atoms were optimized geometrically (C—H = 0.95–0.99 Å) and refined as riding with *U*_iso_(H) = 1.2*U*_eq_(C).

## Supplementary Material

Crystal structure: contains datablock(s) ss069, II, I. DOI: 10.1107/S2056989023000129/ex2064sup1.cif

Structure factors: contains datablock(s) I. DOI: 10.1107/S2056989023000129/ex2064Isup2.hkl

Structure factors: contains datablock(s) II. DOI: 10.1107/S2056989023000129/ex2064IIsup3.hkl

CCDC references: 2234291, 2234292

Additional supporting information:  crystallographic information; 3D view; checkCIF report

## Figures and Tables

**Table 1:** Hydrogen-bond geometry (Å, °) for **I** [Chem scheme1]

*D*—H⋯*A*	*D*—H	H⋯*A*	*D*⋯*A*	*D*—H⋯*A*
C2—H2*A*⋯N4^i^	0.99	2.67	3.5465 (14)	148
C2—H2*B*⋯N4^ii^	0.99	2.68	3.5607 (14)	149
C3—H3*A*⋯N2^iii^	0.99	2.57	3.4149 (13)	143
C7—H7⋯N3^iv^	0.95	2.70	3.5168 (14)	145

Symmetry codes: (i) 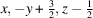 ; (ii) 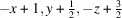 ; (iii) 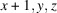 ; (iv) 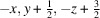 .

**Table 2:** Hydrogen-bond geometry (Å, °) for **II** [Chem scheme1]

*D*—H⋯*A*	*D*—H	H⋯*A*	*D*⋯*A*	*D*—H⋯*A*
C12—H12⋯N3^i^	0.95	2.58	3.3890 (14)	143

Symmetry code: (i) 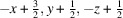 .

**Table 3:** Experimental details

	**I**	**II**
Crystal data		
Chemical formula	C_9_H_12_N_4_O	C_14_H_14_N_4_
*M*_r_	192.23	238.29
Crystal system, space group	Monoclinic, *P*2_1_/*c*	Monoclinic, *P*2_1_/*n*
Temperature (K)	100	100
*a*, *b*, *c* (Å)	5.6889 (3), 8.3058 (4), 20.3063 (8)	15.4187 (2), 4.8130 (1), 15.9993 (3)
β (°)	97.370 (4)	96.115 (2)
*V* (Å^3^)	951.56 (8)	1180.56 (4)
*Z*	4	4
Radiation type	Mo *K*α	Cu *K*α
μ (mm^−1^)	0.09	0.66
Crystal size (mm)	0.26 × 0.16 × 0.14	0.24 × 0.13 × 0.05

Data collection		
Diffractometer	Rigaku FRE+ equipped with VHF Varimax confocal mirrors and an AFC12 goniometer and HyPix 6000 detector	Rigaku FRE+ equipped with VHF Varimax confocal mirrors and an AFC12 goniometer and HyPix 6000 detector
Absorption correction	Analytical (*CrysAlis PRO*; Rigaku OD, 2020 ▸)	Analytical (*CrysAlis PRO*; Rigaku OD, 2020 ▸)
*T*_min_, *T*_max_	0.187, 1.000	0.187, 1.000
No. of measured, independent and observed [*I* > 2σ(*I*)] reflections	47217, 2464, 2280	12295, 2142, 2010
*R*_int_	0.032	0.023
(sin θ/λ)_max_ (Å^−1^)	0.676	0.602

Refinement		
*R*[*F* ^2^ > 2σ(*F* ^2^)], *wR*(*F* ^2^), *S*	0.038, 0.104, 1.05	0.034, 0.094, 1.07
No. of reflections	2464	2142
No. of parameters	127	163
H-atom treatment	H-atom parameters constrained	H-atom parameters constrained
Δρ_max_, Δρ_min_ (e Å^−3^)	0.39, −0.20	0.22, −0.19

Computer programs: *CrysAlis PRO* (Rigaku OD, 2020 ▸), *CrysAlis PRO* (Rigaku OD, 2020), *OLEX2.solve* (Bourhis *et al.*, 2015 ▸), *SHELXL2018/3* (Sheldrick, 2015 ▸) and *OLEX2* (Dolomanov *et al.*, 2009 ▸).

## supplementary crystallographic information

### 4-[(Pyridin-3-yl)diazenyl]morpholine (I) Crystal data

**Table:**

C_9_H_12_N_4_O	*F*(000) = 408
*M**_r_* = 192.23	*D*_x_ = 1.342 Mg m^−^^3^
Monoclinic, *P*2_1_/*c*	Mo *K*α radiation, λ = 0.71075 Å
*a* = 5.6889 (3) Å	Cell parameters from 10944 reflections
*b* = 8.3058 (4) Å	θ = 2.0–36.0°
*c* = 20.3063 (8) Å	µ = 0.09 mm^−^^1^
β = 97.370 (4)°	*T* = 100 K
*V* = 951.56 (8) Å^3^	(cut) irregular block, colourless
*Z* = 4	0.26 × 0.16 × 0.14 mm

### 4-[(Pyridin-3-yl)diazenyl]morpholine (I) Data collection

**Table:**

Rigaku FRE+ equipped with VHF Varimax confocal mirrors and an AFC12 goniometer and HyPix 6000 detector diffractometer	2280 reflections with *I* > 2σ(*I*)
Detector resolution: 10 pixels mm^-1^	*R*_int_ = 0.032
profile data from ω–scans	θ_max_ = 28.7°, θ_min_ = 2.0°
Absorption correction: analytical (CrysAlisPro; Rigaku OD, 2020)	*h* = −7→7
*T*_min_ = 0.187, *T*_max_ = 1.000	*k* = −11→11
47217 measured reflections	*l* = −27→27
2464 independent reflections	

### 4-[(Pyridin-3-yl)diazenyl]morpholine (I) Refinement

**Table:**

Refinement on *F*^2^	Primary atom site location: iterative
Least-squares matrix: full	Hydrogen site location: inferred from neighbouring sites
*R*[*F*^2^ > 2σ(*F*^2^)] = 0.038	H-atom parameters constrained
*wR*(*F*^2^) = 0.104	*w* = 1/[σ^2^(*F*_o_^2^) + (0.0504*P*)^2^ + 0.3578*P*] where *P* = (*F*_o_^2^ + 2*F*_c_^2^)/3
*S* = 1.05	(Δ/σ)_max_ = 0.001
2464 reflections	Δρ_max_ = 0.39 e Å^−^^3^
127 parameters	Δρ_min_ = −0.20 e Å^−^^3^
0 restraints	

### 4-[(Pyridin-3-yl)diazenyl]morpholine (I) Special details

**Table:**

Geometry. All esds (except the esd in the dihedral angle between two l.s. planes) are estimated using the full covariance matrix. The cell esds are taken into account individually in the estimation of esds in distances, angles and torsion angles; correlations between esds in cell parameters are only used when they are defined by crystal symmetry. An approximate (isotropic) treatment of cell esds is used for estimating esds involving l.s. planes.

### 4-[(Pyridin-3-yl)diazenyl]morpholine (I) Fractional atomic coordinates and isotropic or equivalent isotropic displacement parameters (Å^2^)

**Table:**

	*x*	*y*	*z*	*U*_iso_*/*U*_eq_	
C2	0.62623 (19)	0.79887 (13)	0.51038 (5)	0.0239 (2)	
H2A	0.569056	0.840631	0.465513	0.029*	
H2B	0.731480	0.881059	0.533953	0.029*	
C1	0.41701 (18)	0.76924 (14)	0.54796 (5)	0.0245 (2)	
H1A	0.334523	0.872026	0.554175	0.029*	
H1B	0.303268	0.695170	0.522423	0.029*	
C4	0.65470 (18)	0.55810 (12)	0.61131 (5)	0.0220 (2)	
H4A	0.561459	0.464763	0.592211	0.026*	
H4B	0.724736	0.530281	0.657060	0.026*	
C3	0.84953 (18)	0.59792 (14)	0.56928 (5)	0.0240 (2)	
H3A	0.953897	0.681815	0.591964	0.029*	
H3B	0.946831	0.500602	0.564925	0.029*	
C5	0.25737 (17)	0.66881 (12)	0.75890 (5)	0.0195 (2)	
C6	0.05135 (18)	0.76118 (13)	0.75068 (5)	0.0224 (2)	
H6	0.000875	0.814030	0.709820	0.027*	
C7	−0.07817 (18)	0.77375 (13)	0.80398 (5)	0.0248 (2)	
H7	−0.220732	0.834549	0.800057	0.030*	
C8	0.00371 (19)	0.69618 (14)	0.86306 (5)	0.0258 (2)	
H8	−0.086330	0.706044	0.899152	0.031*	
N1	0.50202 (15)	0.69888 (11)	0.61229 (4)	0.02041 (19)	
N2	0.36372 (14)	0.72537 (10)	0.65955 (4)	0.01964 (18)	
N3	0.40585 (14)	0.63392 (10)	0.70942 (4)	0.01969 (18)	
O1	0.75628 (13)	0.65370 (9)	0.50469 (3)	0.02197 (17)	
N4	0.20195 (17)	0.60850 (12)	0.87196 (4)	0.0274 (2)	
C9	0.32349 (18)	0.59528 (13)	0.82007 (5)	0.0239 (2)	
H9	0.463560	0.531802	0.825257	0.029*	

### 4-[(Pyridin-3-yl)diazenyl]morpholine (I) Atomic displacement parameters (Å^2^)

**Table:**

	*U*^11^	*U*^22^	*U*^33^	*U*^12^	*U*^13^	*U*^23^
C2	0.0285 (5)	0.0252 (5)	0.0193 (4)	0.0003 (4)	0.0080 (4)	0.0021 (4)
C1	0.0244 (5)	0.0300 (5)	0.0202 (4)	0.0050 (4)	0.0068 (4)	0.0045 (4)
C4	0.0239 (5)	0.0249 (5)	0.0182 (4)	0.0031 (4)	0.0070 (3)	0.0021 (4)
C3	0.0213 (4)	0.0339 (5)	0.0175 (4)	0.0037 (4)	0.0051 (3)	0.0012 (4)
C5	0.0211 (4)	0.0214 (4)	0.0169 (4)	−0.0034 (3)	0.0054 (3)	−0.0031 (3)
C6	0.0250 (5)	0.0250 (5)	0.0174 (4)	0.0003 (4)	0.0038 (4)	−0.0003 (4)
C7	0.0237 (5)	0.0264 (5)	0.0254 (5)	0.0014 (4)	0.0075 (4)	−0.0040 (4)
C8	0.0306 (5)	0.0286 (5)	0.0206 (5)	−0.0037 (4)	0.0126 (4)	−0.0036 (4)
N1	0.0214 (4)	0.0245 (4)	0.0167 (4)	0.0018 (3)	0.0074 (3)	0.0006 (3)
N2	0.0195 (4)	0.0225 (4)	0.0176 (4)	−0.0012 (3)	0.0048 (3)	−0.0005 (3)
N3	0.0200 (4)	0.0220 (4)	0.0175 (4)	0.0002 (3)	0.0037 (3)	−0.0005 (3)
O1	0.0243 (4)	0.0279 (4)	0.0147 (3)	0.0010 (3)	0.0064 (3)	−0.0008 (3)
N4	0.0344 (5)	0.0304 (5)	0.0185 (4)	0.0003 (4)	0.0073 (3)	0.0015 (3)
C9	0.0253 (5)	0.0265 (5)	0.0203 (5)	0.0017 (4)	0.0048 (4)	0.0000 (4)

### 4-[(Pyridin-3-yl)diazenyl]morpholine (I) Geometric parameters (Å, º)

**Table:**

C2—H2A	0.9900	C5—C6	1.3928 (14)
C2—H2B	0.9900	C5—N3	1.4230 (12)
C2—C1	1.5142 (14)	C5—C9	1.3918 (14)
C2—O1	1.4271 (12)	C6—H6	0.9500
C1—H1A	0.9900	C6—C7	1.3891 (13)
C1—H1B	0.9900	C7—H7	0.9500
C1—N1	1.4559 (12)	C7—C8	1.3886 (15)
C4—H4A	0.9900	C8—H8	0.9500
C4—H4B	0.9900	C8—N4	1.3351 (15)
C4—C3	1.5195 (13)	N1—N2	1.3350 (11)
C4—N1	1.4583 (13)	N2—N3	1.2640 (12)
C3—H3A	0.9900	N4—C9	1.3370 (13)
C3—H3B	0.9900	C9—H9	0.9500
C3—O1	1.4275 (12)		
			
H2A—C2—H2B	108.1	O1—C3—H3B	109.2
C1—C2—H2A	109.5	C6—C5—N3	126.45 (9)
C1—C2—H2B	109.5	C9—C5—C6	118.40 (9)
O1—C2—H2A	109.5	C9—C5—N3	115.07 (9)
O1—C2—H2B	109.5	C5—C6—H6	121.0
O1—C2—C1	110.65 (8)	C7—C6—C5	118.06 (9)
C2—C1—H1A	109.9	C7—C6—H6	121.0
C2—C1—H1B	109.9	C6—C7—H7	120.5
H1A—C1—H1B	108.3	C8—C7—C6	119.05 (10)
N1—C1—C2	108.99 (8)	C8—C7—H7	120.5
N1—C1—H1A	109.9	C7—C8—H8	118.2
N1—C1—H1B	109.9	N4—C8—C7	123.64 (9)
H4A—C4—H4B	108.4	N4—C8—H8	118.2
C3—C4—H4A	110.1	C1—N1—C4	116.08 (8)
C3—C4—H4B	110.1	N2—N1—C1	114.85 (8)
N1—C4—H4A	110.1	N2—N1—C4	123.29 (8)
N1—C4—H4B	110.1	N3—N2—N1	114.09 (8)
N1—C4—C3	108.17 (8)	N2—N3—C5	112.00 (8)
C4—C3—H3A	109.2	C2—O1—C3	109.59 (7)
C4—C3—H3B	109.2	C8—N4—C9	116.83 (9)
H3A—C3—H3B	107.9	C5—C9—H9	118.0
O1—C3—C4	112.02 (8)	N4—C9—C5	124.01 (10)
O1—C3—H3A	109.2	N4—C9—H9	118.0
			
C2—C1—N1—C4	−51.60 (12)	C6—C7—C8—N4	−0.19 (17)
C2—C1—N1—N2	154.23 (9)	C7—C8—N4—C9	−0.74 (17)
C1—C2—O1—C3	−62.24 (10)	C8—N4—C9—C5	1.07 (16)
C1—N1—N2—N3	163.81 (9)	N1—C4—C3—O1	−54.11 (11)
C4—C3—O1—C2	61.98 (11)	N1—N2—N3—C5	178.85 (8)
C4—N1—N2—N3	11.73 (13)	N3—C5—C6—C7	176.15 (9)
C3—C4—N1—C1	50.22 (11)	N3—C5—C9—N4	−177.49 (10)
C3—C4—N1—N2	−158.00 (9)	O1—C2—C1—N1	55.81 (11)
C5—C6—C7—C8	0.81 (16)	C9—C5—C6—C7	−0.52 (15)
C6—C5—N3—N2	15.17 (14)	C9—C5—N3—N2	−168.06 (9)
C6—C5—C9—N4	−0.45 (16)		

### 4-[(Pyridin-3-yl)diazenyl]morpholine (I) Hydrogen-bond geometry (Å, º)

**Table:**

*D*—H···*A*	*D*—H	H···*A*	*D*···*A*	*D*—H···*A*
C2—H2*A*···N4^i^	0.99	2.67	3.5465 (14)	148
C2—H2*B*···N4^ii^	0.99	2.68	3.5607 (14)	149
C3—H3*A*···N2^iii^	0.99	2.57	3.4149 (13)	143
C7—H7···N3^iv^	0.95	2.70	3.5168 (14)	145

Symmetry codes: (i) *x*, −*y*+3/2, *z*−1/2; (ii) −*x*+1, *y*+1/2, −*z*+3/2; (iii) *x*+1, *y*, *z*; (iv) −*x*, *y*+1/2, −*z*+3/2.

### 1-[(Pyridin-3-yl)diazenyl]-1,2,3,4-tetrahydroquinoline (II) Crystal data

**Table:**

C_14_H_14_N_4_	*F*(000) = 504
*M**_r_* = 238.29	*D*_x_ = 1.341 Mg m^−^^3^
Monoclinic, *P*2_1_/*n*	Cu *K*α radiation, λ = 1.54178 Å
*a* = 15.4187 (2) Å	Cell parameters from 7895 reflections
*b* = 4.8130 (1) Å	θ = 2.0–36.0°
*c* = 15.9993 (3) Å	µ = 0.66 mm^−^^1^
β = 96.115 (2)°	*T* = 100 K
*V* = 1180.56 (4) Å^3^	(cut) irregular block, colourless
*Z* = 4	0.24 × 0.13 × 0.05 mm

### 1-[(Pyridin-3-yl)diazenyl]-1,2,3,4-tetrahydroquinoline (II) Data collection

**Table:**

Rigaku FRE+ equipped with VHF Varimax confocal mirrors and an AFC12 goniometer and HyPix 6000 detector diffractometer	2010 reflections with *I* > 2σ(*I*)
Detector resolution: 10 pixels mm^-1^	*R*_int_ = 0.023
profile data from ω–scans	θ_max_ = 68.2°, θ_min_ = 5.8°
Absorption correction: analytical (CrysAlisPro; Rigaku OD, 2020)	*h* = −18→17
*T*_min_ = 0.187, *T*_max_ = 1.000	*k* = −5→5
12295 measured reflections	*l* = −19→19
2142 independent reflections	

### 1-[(Pyridin-3-yl)diazenyl]-1,2,3,4-tetrahydroquinoline (II) Refinement

**Table:**

Refinement on *F*^2^	Primary atom site location: iterative
Least-squares matrix: full	Hydrogen site location: inferred from neighbouring sites
*R*[*F*^2^ > 2σ(*F*^2^)] = 0.034	H-atom parameters constrained
*wR*(*F*^2^) = 0.094	*w* = 1/[σ^2^(*F*_o_^2^) + (0.0501*P*)^2^ + 0.4022*P*] where *P* = (*F*_o_^2^ + 2*F*_c_^2^)/3
*S* = 1.07	(Δ/σ)_max_ = 0.001
2142 reflections	Δρ_max_ = 0.22 e Å^−^^3^
163 parameters	Δρ_min_ = −0.19 e Å^−^^3^
0 restraints	

### 1-[(Pyridin-3-yl)diazenyl]-1,2,3,4-tetrahydroquinoline (II) Special details

**Table:**

Geometry. All esds (except the esd in the dihedral angle between two l.s. planes) are estimated using the full covariance matrix. The cell esds are taken into account individually in the estimation of esds in distances, angles and torsion angles; correlations between esds in cell parameters are only used when they are defined by crystal symmetry. An approximate (isotropic) treatment of cell esds is used for estimating esds involving l.s. planes.

### 1-[(Pyridin-3-yl)diazenyl]-1,2,3,4-tetrahydroquinoline (II) Fractional atomic coordinates and isotropic or equivalent isotropic displacement parameters (Å^2^)

**Table:**

	*x*	*y*	*z*	*U*_iso_*/*U*_eq_	
C1	0.33530 (7)	0.4875 (2)	0.29703 (7)	0.0231 (3)	
H1	0.279699	0.515484	0.266503	0.028*	
C2	0.40405 (7)	0.6536 (2)	0.27811 (7)	0.0233 (3)	
H2	0.394906	0.793635	0.236253	0.028*	
C3	0.48588 (7)	0.6140 (2)	0.32051 (7)	0.0220 (3)	
H3	0.533766	0.726560	0.308860	0.026*	
C4	0.49636 (7)	0.4040 (2)	0.38099 (7)	0.0190 (3)	
C5	0.42285 (7)	0.2529 (2)	0.39706 (7)	0.0222 (3)	
H5	0.429669	0.115020	0.439742	0.027*	
C6	0.72221 (7)	0.1768 (2)	0.51239 (7)	0.0207 (3)	
H6A	0.709233	−0.014174	0.491712	0.025*	
H6B	0.677731	0.229166	0.549830	0.025*	
C7	0.81190 (7)	0.1837 (2)	0.56141 (7)	0.0226 (3)	
H7A	0.815446	0.040449	0.606052	0.027*	
H7B	0.821715	0.367432	0.588596	0.027*	
C8	0.88195 (7)	0.1301 (2)	0.50322 (7)	0.0206 (3)	
H8A	0.940339	0.144430	0.535296	0.025*	
H8B	0.875328	−0.060116	0.479846	0.025*	
C9	0.87424 (7)	0.3391 (2)	0.43253 (7)	0.0183 (3)	
C10	0.79318 (7)	0.4562 (2)	0.40429 (7)	0.0181 (2)	
C11	0.78684 (7)	0.6578 (2)	0.34059 (7)	0.0203 (3)	
H11	0.732038	0.740036	0.322463	0.024*	
C12	0.86059 (7)	0.7368 (2)	0.30415 (7)	0.0219 (3)	
H12	0.856037	0.871900	0.260628	0.026*	
C13	0.94119 (7)	0.6197 (2)	0.33081 (7)	0.0223 (3)	
H13	0.991636	0.673085	0.305469	0.027*	
C14	0.94718 (7)	0.4242 (2)	0.39480 (7)	0.0210 (3)	
H14	1.002478	0.346140	0.413404	0.025*	
N1	0.34340 (6)	0.2893 (2)	0.35634 (6)	0.0251 (2)	
N2	0.57576 (6)	0.32558 (19)	0.42816 (6)	0.0196 (2)	
N3	0.64124 (6)	0.43689 (19)	0.39911 (6)	0.0189 (2)	
N4	0.71783 (6)	0.36903 (19)	0.44088 (6)	0.0187 (2)	

### 1-[(Pyridin-3-yl)diazenyl]-1,2,3,4-tetrahydroquinoline (II) Atomic displacement parameters (Å^2^)

**Table:**

	*U*^11^	*U*^22^	*U*^33^	*U*^12^	*U*^13^	*U*^23^
C1	0.0221 (5)	0.0212 (6)	0.0257 (6)	0.0030 (5)	0.0019 (4)	−0.0037 (5)
C2	0.0265 (6)	0.0207 (6)	0.0233 (6)	0.0034 (5)	0.0050 (5)	0.0010 (5)
C3	0.0227 (5)	0.0193 (6)	0.0248 (6)	−0.0010 (4)	0.0066 (4)	−0.0001 (5)
C4	0.0207 (5)	0.0174 (5)	0.0194 (5)	0.0011 (4)	0.0048 (4)	−0.0039 (4)
C5	0.0232 (6)	0.0188 (6)	0.0252 (6)	0.0005 (5)	0.0048 (4)	0.0007 (5)
C6	0.0235 (6)	0.0191 (6)	0.0200 (5)	−0.0015 (4)	0.0052 (4)	0.0030 (4)
C7	0.0257 (6)	0.0220 (6)	0.0198 (5)	−0.0017 (5)	0.0016 (4)	0.0039 (5)
C8	0.0224 (5)	0.0157 (6)	0.0235 (6)	−0.0009 (4)	0.0012 (4)	0.0017 (4)
C9	0.0228 (5)	0.0129 (5)	0.0193 (5)	−0.0013 (4)	0.0023 (4)	−0.0028 (4)
C10	0.0209 (5)	0.0155 (5)	0.0180 (5)	−0.0033 (4)	0.0035 (4)	−0.0029 (4)
C11	0.0222 (5)	0.0178 (6)	0.0206 (5)	−0.0004 (4)	0.0008 (4)	0.0001 (4)
C12	0.0282 (6)	0.0190 (6)	0.0188 (6)	−0.0034 (5)	0.0033 (4)	0.0018 (4)
C13	0.0235 (6)	0.0210 (6)	0.0234 (6)	−0.0033 (5)	0.0076 (4)	−0.0013 (5)
C14	0.0210 (5)	0.0175 (6)	0.0250 (6)	0.0010 (4)	0.0041 (4)	−0.0021 (4)
N1	0.0225 (5)	0.0215 (5)	0.0314 (6)	−0.0004 (4)	0.0031 (4)	0.0000 (4)
N2	0.0204 (5)	0.0177 (5)	0.0213 (5)	−0.0014 (4)	0.0052 (4)	−0.0014 (4)
N3	0.0199 (5)	0.0171 (5)	0.0199 (5)	−0.0007 (4)	0.0033 (4)	−0.0020 (4)
N4	0.0192 (5)	0.0181 (5)	0.0188 (5)	−0.0011 (4)	0.0022 (4)	0.0021 (4)

### 1-[(Pyridin-3-yl)diazenyl]-1,2,3,4-tetrahydroquinoline (II) Geometric parameters (Å, º)

**Table:**

C1—H1	0.9500	C7—C8	1.5212 (15)
C1—C2	1.3865 (16)	C8—H8A	0.9900
C1—N1	1.3422 (16)	C8—H8B	0.9900
C2—H2	0.9500	C8—C9	1.5090 (15)
C2—C3	1.3807 (16)	C9—C10	1.4013 (15)
C3—H3	0.9500	C9—C14	1.3934 (15)
C3—C4	1.3967 (16)	C10—C11	1.4030 (15)
C4—C5	1.3935 (16)	C10—N4	1.4189 (13)
C4—N2	1.4192 (14)	C11—H11	0.9500
C5—H5	0.9500	C11—C12	1.3851 (16)
C5—N1	1.3363 (15)	C12—H12	0.9500
C6—H6A	0.9900	C12—C13	1.3895 (16)
C6—H6B	0.9900	C13—H13	0.9500
C6—C7	1.5159 (15)	C13—C14	1.3864 (16)
C6—N4	1.4674 (14)	C14—H14	0.9500
C7—H7A	0.9900	N2—N3	1.2737 (13)
C7—H7B	0.9900	N3—N4	1.3341 (12)
			
C2—C1—H1	118.4	H8A—C8—H8B	108.2
N1—C1—H1	118.4	C9—C8—C7	109.95 (9)
N1—C1—C2	123.27 (10)	C9—C8—H8A	109.7
C1—C2—H2	120.2	C9—C8—H8B	109.7
C3—C2—C1	119.54 (11)	C10—C9—C8	120.41 (10)
C3—C2—H2	120.2	C14—C9—C8	121.19 (10)
C2—C3—H3	120.9	C14—C9—C10	118.39 (10)
C2—C3—C4	118.19 (10)	C9—C10—C11	120.21 (10)
C4—C3—H3	120.9	C9—C10—N4	119.30 (10)
C3—C4—N2	126.19 (10)	C11—C10—N4	120.49 (10)
C5—C4—C3	117.99 (10)	C10—C11—H11	120.0
C5—C4—N2	115.82 (10)	C12—C11—C10	119.91 (10)
C4—C5—H5	117.9	C12—C11—H11	120.0
N1—C5—C4	124.27 (11)	C11—C12—H12	119.8
N1—C5—H5	117.9	C11—C12—C13	120.47 (10)
H6A—C6—H6B	108.1	C13—C12—H12	119.8
C7—C6—H6A	109.5	C12—C13—H13	120.4
C7—C6—H6B	109.5	C14—C13—C12	119.27 (10)
N4—C6—H6A	109.5	C14—C13—H13	120.4
N4—C6—H6B	109.5	C9—C14—H14	119.1
N4—C6—C7	110.68 (9)	C13—C14—C9	121.74 (10)
C6—C7—H7A	109.6	C13—C14—H14	119.1
C6—C7—H7B	109.6	C5—N1—C1	116.69 (10)
C6—C7—C8	110.35 (9)	N3—N2—C4	111.49 (9)
H7A—C7—H7B	108.1	N2—N3—N4	114.08 (9)
C8—C7—H7A	109.6	C10—N4—C6	122.45 (9)
C8—C7—H7B	109.6	N3—N4—C6	120.64 (9)
C7—C8—H8A	109.7	N3—N4—C10	116.17 (9)
C7—C8—H8B	109.7		
			
C1—C2—C3—C4	0.55 (17)	C9—C10—C11—C12	1.46 (16)
C2—C1—N1—C5	−1.03 (17)	C9—C10—N4—C6	5.22 (15)
C2—C3—C4—C5	−2.17 (16)	C9—C10—N4—N3	−164.91 (9)
C2—C3—C4—N2	177.24 (10)	C10—C9—C14—C13	0.03 (16)
C3—C4—C5—N1	2.38 (17)	C10—C11—C12—C13	−0.64 (17)
C3—C4—N2—N3	−11.45 (15)	C11—C10—N4—C6	−174.70 (10)
C4—C5—N1—C1	−0.77 (17)	C11—C10—N4—N3	15.17 (15)
C4—N2—N3—N4	−179.67 (8)	C11—C12—C13—C14	−0.47 (17)
C5—C4—N2—N3	167.97 (10)	C12—C13—C14—C9	0.78 (17)
C6—C7—C8—C9	56.49 (12)	C14—C9—C10—C11	−1.15 (16)
C7—C6—N4—C10	23.41 (14)	C14—C9—C10—N4	178.93 (9)
C7—C6—N4—N3	−166.89 (9)	N1—C1—C2—C3	1.13 (18)
C7—C8—C9—C10	−28.76 (14)	N2—C4—C5—N1	−177.08 (10)
C7—C8—C9—C14	150.10 (10)	N2—N3—N4—C6	1.30 (14)
C8—C9—C10—C11	177.73 (10)	N2—N3—N4—C10	171.63 (9)
C8—C9—C10—N4	−2.19 (15)	N4—C6—C7—C8	−53.93 (12)
C8—C9—C14—C13	−178.84 (10)	N4—C10—C11—C12	−178.62 (10)

### 1-[(Pyridin-3-yl)diazenyl]-1,2,3,4-tetrahydroquinoline (II) Hydrogen-bond geometry (Å, º)

**Table:**

*D*—H···*A*	*D*—H	H···*A*	*D*···*A*	*D*—H···*A*
C12—H12···N3^i^	0.95	2.58	3.3890 (14)	143

Symmetry code: (i) −*x*+3/2, *y*+1/2, −*z*+1/2.
